# Draft Genome Sequence of a Colistin-Resistant *Klebsiella pneumoniae* Clinical Strain Carrying the *bla*_NDM-1_ Carbapenemase Gene

**DOI:** 10.1128/genomeA.01654-16

**Published:** 2017-02-16

**Authors:** Zhihong Yao, Yu Feng, Ji Lin, Zhiyong Zong

**Affiliations:** a Center of Infectious Diseases, West China Hospital, Sichuan University, Chengdu, China; b Division of Infectious Diseases, State Key Laboratory of Biotherapy, Chengdu, China; c Department of Infection Control, West China Hospital, Sichuan University, Chengdu, China; d Faculty of Pharmacology and Medical Technology, Hanzhong Vocational and Technical College, Hanzhong, Shaanxi, China

## Abstract

*Klebsiella pneumoniae* strain WCHKP1845, recovered from the sputum of a patient with pneumonia, was resistant to colistin and carried the carbapenemase gene *bla*_NDM-1_. Here, we report its 5.4-Mb draft genome sequence, comprising 140 contigs with an average 57.33% G+C content. The genome contains 5,118 coding sequences and 88 tRNA genes.

## GENOME ANNOUNCEMENT

*Klebsiella pneumoniae* is a major pathogen of human infections (1). *K. pneumoniae* strain WCHKP1845 was recovered from the sputum of a male patient with pneumonia in the Respiratory Intensive Care Unit at West China Hospital, Chengdu, Western China, in May 2016. Strain WCHKP1845 is resistant to meropenem (MIC, 64 μg/mL) and colistin (MIC, 16 μg/mL). Screening for the acquired carbapenem-hydrolyzing β-lactamase (carbapenemase) genes *bla*_GES_ (including noncarbapenemase variants), *bla*_IMP_, *bla*_IMI_, *bla*_KPC_, *bla*_NDM_, *bla*_OXA-48_-like, and *bla*_VIM_ was performed using PCR as described previously (2–5). Strain WCHKP1845 was positive for *bla*_NDM_ and was therefore subjected to whole-genome sequencing.

The QIAamp DNA minikit (Qiagen, Hilden, Germany) was used to prepare genomic DNA from strain WCHKP1845, which was then sequenced using the HiSeq X10 Sequencer (Illumina, San Diego, CA, USA) with the 150-bp paired-end protocol and 200× coverage. A total of 1.5-Gb clean bases and 5,013,767 reads were generated, which were assembled into 140 contigs (76 contigs ≥1,000 bp in length; *N*_50_, 334,879 bp) with a 57.33% G+C content using SPAdes version 3.9 (6). The genome size was about 5.4 Mb, which was annotated using Prokka version 1.11 (7). The genome of strain WCHKP1845 contained 5,118 coding sequences and 88 tRNA genes. The multilocus sequence typing (MLST) tool of the Center for Genomic Epidemiology (http://genomicepidemiology.org) was used to assign strain WCHKP1845 to a sequence type (ST) based on the genome sequence. Strain WCHKP1845 belonged to ST1 (*gapA*, *infB*, *mdh*, *pgi*, *phoE*, *rpoB*, and *tonB*; 4-4-1-1-7-4-10). *K. pneumoniae* of ST1 has been found in China (8) and Europe (Spain) (9) (http://bigsdb.pasteur.fr/klebsiella/klebsiella.html).

Antimicrobial resistance genes were predicted using ResFinder from the Center for Genomic Epidemiology. Strain WCHKP1845 had the carbapenemase gene *bla*_NDM-1_ and a few other antimicrobial resistance genes, including *bla*_TEM-1a_ and *bla*_SHV-1_ (mediating resistance to penicillins), *fosA* (to fosfomycin), *oqxA* and* oqxB* (to quinolones), *strA* and* strB* (to aminoglycosides), *sul2* (to sulfonamides), and *tet*_(A)_ (to tetracycline). Of note, although strain WCHKP1845 was resistant to colistin, it did not carry the plasmid-borne colistin-resistant genes *mcr-1* and *mcr-2*. The interruption of *mgrB*, which encodes a negative-feedback regulator of the PhoQ-PhoP two-component system by insertion sequences, has been reported as the common mechanism of colistin resistance in carbapenem-resistant *K. pneumoniae* (10). However, the *mgrB* gene of strain WCHKP1845 is intact. Therefore, the mechanism of colistin resistance in strain WCHKP1845 remains unclear at present and warrants further investigation. PlasmidFinder from the Center for Genomic Epidemiology predicted that strain WCHKP1845 carried five plasmid replicons, including IncQ1, IncR, IncX3, and two IncFII(K). *bla*_NDM-1_ is located on a 48,124 bp-contig that contains the IncX3 replicon, suggesting that *bla*_NDM-1_ is carried by an IncX3 plasmid.

### Accession number(s).

This whole-genome shotgun project has been deposited at DDBJ/EMBL/GenBank under the accession number MPOD00000000. The version described in this paper is the first version, MPOD01000000.

## References

[1] Broberg CA, Palacios M, Miller VL 2014 *Klebsiella*: a long way to go towards understanding this enigmatic jet-setter. F1000Prime Rep 6:64. doi:10.12703/P6-64. 25165563 PMC4126530

[2] Zong Z, Zhang X 2013 *bla*_NDM-1_-carrying *Acinetobacter* *johnsonii* detected in hospital sewage. J Antimicrob Chemother 68:1007–1010. doi:10.1093/jac/dks505. 23288403

[3] Mendes RE, Kiyota KA, Monteiro J, Castanheira M, Andrade SS, Gales AC, Pignatari AC, Tufik S 2007 Rapid detection and identification of metallo-β-lactamase-encoding genes by multiplex real-time PCR assay and melt curve analysis. J Clin Microbiol 45:544–547. doi:10.1128/JCM.01728-06. 17093019 PMC1829038

[4] Poirel L, Le Thomas I, Naas T, Karim A, Nordmann P 2000 Biochemical sequence analyses of GES-1, a novel class A extended-spectrum β-lactamase, and the class 1 integron In*52* from *Klebsiella pneumoniae*. Antimicrob Agents Chemother 44:622–632. doi:10.1128/AAC.44.3.622-632.2000. 10681329 PMC89737

[5] Bradford PA, Bratu S, Urban C, Visalli M, Mariano N, Landman D, Rahal JJ, Brooks S, Cebular S, Quale J 2004 Emergence of carbapenem-resistant *Klebsiella* species possessing the class A carbapenem-hydrolyzing KPC-2 and inhibitor-resistant TEM-30 β-lactamases in New York City. Clin Infect Dis 39:55–60. doi:10.1086/421495. 15206053

[6] Bankevich A, Nurk S, Antipov D, Gurevich AA, Dvorkin M, Kulikov AS, Lesin VM, Nikolenko SI, Pham S, Prjibelski AD, Pyshkin AV, Sirotkin AV, Vyahhi N, Tesler G, Alekseyev MA, Pevzner PA 2012 SPAdes: a new genome assembly algorithm and its applications to single-cell sequencing. J Comput Biol 19:455–477. doi:10.1089/cmb.2012.0021. 22506599 PMC3342519

[7] Seemann T 2014 Prokka: rapid prokaryotic genome annotation. Bioinformatics 30:2068–2069. doi:10.1093/bioinformatics/btu153. 24642063

[8] Guo C, Yang X, Wu Y, Yang H, Han Y, Yang R, Hu L, Cui Y, Zhou D 2015 MLST-based inference of genetic diversity and population structure of clinical *Klebsiella pneumoniae*, China. Sci Rep 5:7612. doi:10.1038/srep07612. 25556771 PMC5154592

[9] Diancourt L, Passet V, Verhoef J, Grimont PA, Brisse S 2005 Multilocus sequence typing of *Klebsiella pneumoniae* nosocomial isolates. J Clin Microbiol 43:4178–4182. doi:10.1128/JCM.43.8.4178-4182.2005. 16081970 PMC1233940

[10] Cannatelli A, Giani T, D’Andrea MM, Di Pilato V, Arena F, Conte V, Tryfinopoulou K, Vatopoulos A, Rossolini GM, COLGRIT Study Group 2014 MgrB inactivation is a common mechanism of colistin resistance in KPC-producing *Klebsiella pneumoniae* of clinical origin. Antimicrob Agents Chemother 58:5696–5703. doi:10.1128/AAC.03110-14. 25022583 PMC4187966

